# Cohort profile: the provincial opioid agonist treatment cohort in Ontario, Canada

**DOI:** 10.1007/s10654-025-01202-3

**Published:** 2025-01-27

**Authors:** Kristen A. Morin, Mark R. Tatangelo, Shreedhar Acharya, David C. Marsh

**Affiliations:** 1 Health Sciences North Research Institute, Sudbury, ON Canada; 2 ICES North (Institute for Clinical and Evaluative Sciences), Sudbury, ON Canada; 3 Northern Ontario School of Medicine University, Sudbury, ON P3E 2C6 Canada

**Keywords:** Observational cohort study, Opioid agonist treatment, Addiction medicine, Longitudinal study, Administrative data

## Abstract

**Background:**

Opioid Agonist Treatment (OAT) is the most effective intervention for opioid use disorder (OUD), but retention has decreased due to increasingly potent drugs like fentanyl. This cohort can be used retrospectively to observe trends in service utilization, healthcare integration, healthcare costs and patient outcomes. It also facilitates the design of observational studies to mimic a prospective design.

**Methods:**

This study used linked administrative data from ICES to create a cohort of 137,035 individuals who received at least one prescription of methadone or buprenorphine/naloxone between 2014 and 2022. Data were linked using de-identified personal health numbers. Variables included age, sex, rurality, income, homelessness, and mental health conditions. Regional differences in OAT use, retention, and mortality were analyzed.

**Results:**

Of the cohort, 56.1% began OAT after 2014. Southern Ontario participants more often started on methadone (53.2%), while Northern Ontario patients favored buprenorphine/naloxone (62.7%). Northern patients were younger, more likely to be female, live in rural areas, and face homelessness. The death rate was higher in Southern Ontario (22.1%) than in Northern Ontario (13.2%). Retention declined over time, with 73.4% of patients remaining in treatment at the study's end.

**Conclusions:**

The findings highlight regional disparities in OAT delivery and emphasize the need for region-specific strategies, particularly in rural areas, to improve retention and reduce mortality.

## Study Setting

Opioid use is a significant public health issue linked to morbidity and mortality [1]. Between 2016 and 2023, Canada faced a severe public health crisis with over 40,000 deaths attributable to the toxic drug supply [2]. Ontario alone accounted for approximately 25% of these fatalities, with 10,900 deaths reported during this period. Northern Ontario, despite representing less than 10% of the population, experienced about 15% of these deaths, totaling 1411 fatalities [3]. These regional disparities underscore a troubling trend: areas with smaller populations are experiencing disproportionately high numbers of drug-related deaths [4, 5].

People who use opioids (PWUO) experience co-occurring mental disorders [6–8], infections [9–11], and chronic illnesses [12], compounded by elevated rates of poverty [13] and homelessness [13–15]. Despite the issues they face, PWUO encounter significant issues accessing care. As a result, emergency departments (ED) are critical in managing complications related to substance use, often serving as the primary point of contact for individuals in crisis [16]. The high demand for ED services not only contributes to a significant number of costly visits but leads to longer wait times for other medical conditions, exacerbating the overall strain on healthcare resources [17, 18].

Opioid Agonist Treatment (OAT) is the intervention with the best evidence for OUD. Studies have found that low coverage of OAT leads to higher rates of deaths and overdoses [5]. Moreover, population-level treatment retention has been decreasing due to the potency of the drug supply [19] inhibiting the effectiveness of OAT [20, 21]. Understanding OAT in the era of fentanyl is crucial to continue to improve the system of care for PWUO and to reduce deaths.

Despite the large body of research on people with PWUO, there are limited RCTs and population-level studies to address the high morbidity and mortality unique to the era of fentanyl and its analogs. PWUO are unlikely to participate in conventional randomized control trials (RCT) or complete population-level surveys [22]. Using observational data fills this gap and allows researchers to replicate the structure of RCTs at a population level. This entails establishing eligibility criteria according to data available at baseline and carefully defining treatment strategies. The emulation of an RCT yields the same effect estimates (except for random variability) as target trials [23–25].

This cohort can be used retrospectively to observe trends in service utilization, healthcare integration, healthcare costs and patient outcomes. It also facilitates the design of observational studies to mimic a prospective design. Our approach, built on health administrative database linkages, can deliver exceptional value, with greater external validity than a randomized controlled trial at a fraction of the cost. Additionally, it serves as the groundwork for evaluative studies concerning the costs and benefits of OAT across all regions of Ontario. Our OUD cohort, therefore, presents a pivotal opportunity to assess the impact of these healthcare system changes amidst an unprecedented public health emergency. We secured funding for this cohort from the Northern Ontario Academic Medicine Association (NOAMA) (10). In addition to health care services use, researchers may also use these data using analytic tools that provide more sensitive instruments to detect treatment differences among patients with OUD.

## Data collection

The Provincial Opioid Agonist Treatment Cohort in Ontario, Canada was established in 2019 to evaluate treatment and health service outcomes for people with OUD who are engaged in OAT. This cohort is built using linked population-level administrative databases in ICES. ICES is an independent, non-profit organization, that extracts health informatics for population-wide health outcomes research in Ontario, Canada [26]. We have on hand several linked provincial databases which include all Ontario residents with an indication of OUD from 01/01/2012 to 08/30/2023 (n = 137,035). ICES has deterministically linked the databases on an individual level using a de-identified personal health number.

In Ontario Canada, a single universal payer of health care system, the Ontario Health Insurance Plan (OHIP) records all health care records in a central secure repository as the payer of health care services^37^. The Discharge Abstract Database (DAD) measuring hospital discharge data^36^, the Narcotics Monitoring System database with all narcotic prescriptions^38^ the National Ambulatory Care Reporting System (NACRS)^39^ and the Registered Persons (RPDB) database containing vital statistics updated at each health card renewal (every 5 years or each address change) were provided to the study team by ICES. The datasets were deterministically linked based on unique personal health numbers used to record event-level data within the health care system which are recorded in each database. In addition to these datasets, derived standardized tools are available including validated comorbidity instruments for many chronic and acute conditions (citations), standardized health care costing instruments (citation), and socioeconomic data derived from area level census data including the Ontario Marginalization Index, Index of Rurality, and neighborhood income quintile [27].

### Baseline measures

Covariates include demographic and clinical variables. Demographic information included: age, biological sex assigned at birth (male or female), geographical location, rurality, index of remoteness [28] and rurality index of Ontario [29]income, and homelessness. Geographical locations as defined by Local Health Integration Network (LHIN) (15). LHINs are regional health authorities responsible for regional administration of public healthcare services in Ontario. Ontario had 14 LHINs which provided hospital and community-based care to all residents within their geographical boundaries, Northern geographic indicators were defined patient residence in LHIN 13 or 14 (15).

### Health service use and comorbidities

The DAD contains information on all admitted patient services at all hospitals and the NACRS contains information on all ambulatory care in hospitals including the ED.^42^For acute care, the resource intensity weight, comorbidity code, and the Case Mix Group Plus code^43,44^enable researchers to estimate daily costs per hospital stay or ED visit. The Narcotics Monitoring System (NMS) database includes information on narcotic medications dispensed by the pharmacies in the province; it provides a unique opportunity to estimate the province-wide spending on prescription medications for SUD and co-existing conditions. The NOSM The Ontario Health Insurance Plan (OHIP) database contains information on all billable physician services and diagnosis codes enabling the account of any physician-based service as an outcome or exposure in our models. With these linked databases we can consider key comorbidities among PWOUD including mental disorders, co-occurring substance use disorders, physical comorbidities and infections. We can also a patient’s health service use journey across the continuum of health services including primary care and psychiatry visits, ED and hospitalizations related and unrelated to substance use.

### OAT prescriber characteristics

The characteristics of OAT prescribers include age, sex, and graduation year. Additionally, whether the prescriber completed their education within Canada, the location of practice and practice size, specialty and if that physician is practicing in a rural or urban area.

### Longitudinal follow-up measures

Key measures of longitudinal follow-up of OAT indicators include OAT medication (methadone, buprenorphine/naloxone, extended-release oral morphine or extended-release subcutaneous buprenorphine). The longitudinal follow-up allows for the identification and quantification of OAT services over time, providing critical time-to-event data for patient outcomes with validity and reliability. Study follow-up is observational from clinical interactions; therefore, patients are considered lost to follow-up if they discontinue treatment or die defined as: no clinical visits within 1-year of the last clinical visit. The days of patient follow-up was 1465.84 (1006.45) measured from the initial OAT contact to the conclusion of the study follow-up Fig. 2.

## Cohort demographics and OAT engagement

**Fig. 1:**
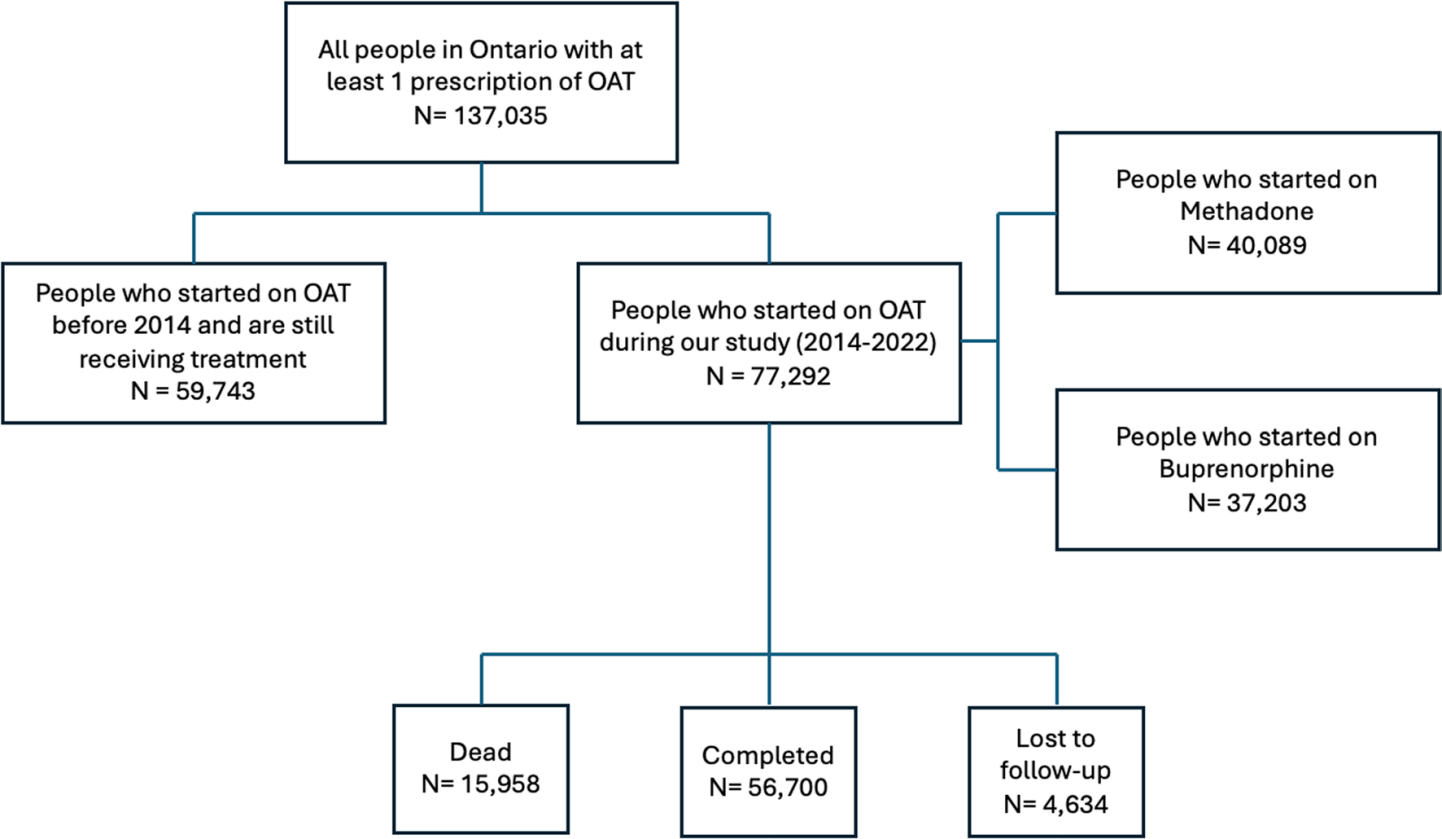
Distribution of OAT patients in Ontario

A total of 137,035 people had at least one prescription of methadone or buprenorphine/naloxone in Ontario between 2014 and 2022. 60,140 (43.9%) people started on OAT before 2014 and were still retained in treatment at the end of our follow-up and 76,890 (56.1%) started on OAT after 2014 (incident new user cohort). Of the incident new users, 64,479 (83.4%) resided in Southern Ontario and 12,813 (16.6%) in Northern Ontario. 34,370 (53.2%) of people living in Southern Ontario started on Methadone in their initial OAT episode and 28,647 (46.8%) on Buprenorphine/naloxone. 4573 (37.3%) of people living in Southern Ontario started on Methadone in their initial OAT episode and 7355 (62.7%) on Buprenorphine/naloxone. Results are presented in Fig. 1.

The mean age of patients in Southern Ontario was 43.75 (SD = 17.65) compared to 37.41 (SD = 14.38) in Northern Ontario. Females comprised 38.2% of the cohort for Southern Ontario and 44.3% in Northern Ontario. Geographic representation varied significantly, with Erie St. Clair (10.3%) and Hamilton Niagara Hamilton and Brant (14.0%) having the highest number of people on OAT in the province. Rurality was also significantly different, with 52.2% of Northern Ontario patients living in rural areas compared to 10.9% in Southern Ontario. Overall, 26.6% of patients reported being homeless, and the death rate was higher in Southern Ontario (22.1%) compared to Northern Ontario (13.2%). The cohort experienced a 6.0% loss to follow-up, with 73.4% still retained at the end of the study period. The results are presented in Table 1.

**Table 1:** Demographic characteristics in the calendar year of cohort entry

	Ontario Total	Southern Ontario	Northern Ontario	SMD
	N = 76,890	N = 64,623 (84.0%)	N = 12,267 (16.0%)	
	77,292	64,479 (83.4)	12,813 (16.6)	0.39
Age (mean (SD))	42.7 (17.32)	43.75 (17.65)	37.41 (14.38)	
Sex (%)				0.182
F	30,327 (39.2)	24,652 (38.2)	5675 (44.3)	
M	46,338 (60.0)	39,200 (60.8)	7138 (55.7)	
X	627 (0.8)	627 (1.0)	0 (0.0)	
Location of residence (%)				3.751
Erie St. Clair	7687 (10.3)	7687 (12.5)	0 (0.0)	
South West	5885 (7.9)	5885 (9.5)	0 (0.0)	
Waterloo Wellington	3710 (5.0)	3710 (6.0)	0 (0.0)	
Hamilton Niagara Hamilton and Brant	10,453 (14.0)	10,453 (16.9)	0 (0.0)	
Central West	3117 (4.2)	3117 (5.1)	0 (0.0)	
Mississauga	3292 (4.4)	3292 (5.3)	0 (0.0)	
Toronto Central	4577 (6.1)	4577 (7.4)	0 (0.0)	
Central	4542 (6.1)	4542 (7.4)	0 (0.0)	
Central East	7068 (9.5)	7068 (11.5)	0 (0.0)	
South East	3448 (4.6)	3448 (5.6)	0 (0.0)	
Champlain	4762 (6.4)	4762 (7.7)	0 (0.0)	
North Simcoe Muskoka	3156 (4.2)	3156 (5.1)	0 (0.0)	
North East	6957 (9.3)	0 (0.0)	6957 (54.3)	
North West	5856 (7.9)	0 (0.0)	5856 (45.7)	
Income quintile (mean (SD))	2.46 (1.49)	2.53 (1.48)	2.13 (1.45)	0.272
Rural (%)				0/925
No	61,332 (79.4)	54,644 (84.7)	6688 (52.2)	
Yes	13,163 (17.0)	7038 (10.9)	6125 (47.8)	
missing	66,803 (82.3)	59,632 (87.9)	7171 (54.0)	
Rurality Index of Ontario (mean (SD))	11.67 (18.46)	9.73 (14.11)	21.43 (30.67)	0.49
Index of Remoteness (mean (SD))	0.15 (0.16)	0.10 (0.07)	0.43 (0.19)	2.342
Homeless (mean (SD))	20,593 (26.6)	17,216 (26.7)	3377 (26.4)	0.008
Death (mean (SD))	15,958 (20.6)	14,267 (22.1)	1691 (13.6)	0.238
Lost to follow up (mean (SD))	4634 (6.0)	3999 (6.2)	635 (5.0)	0.024
Still retained at the end of study period (mean (SD))	56,700 (73.4)	46,213 (71.7)	10,487 (81.8)	0.228

*n = number

*mean = mean per year

*SD = standard deviation

*SMD = standardized mean difference

Overall, 42.1% of patients were on buprenorphine/naloxone as a first-time medication, with a statistically significantly higher proportion in Northern Ontario (57.0%) compared to Southern Ontario (39.1%). Methadone was prescribed to 44.8% of the cohort as a first time OAT, with statistically significantly higher use in Southern Ontario (46.6%) compared to Northern Ontario (35.7%). The mean days retained during the first OAT attempt was 166.04 days (SD 297.25), with Northern Ontario exhibiting lower retention (133.77 days) compared to Southern Ontario (172.45 days). Buprenorphine/naloxone retention was significantly higher in Northern Ontario (206.57 days) than in Southern Ontario (94.25 days). For methadone, Southern Ontario saw longer retention (243.05 days) compared to Northern Ontario (173.35 days).

The mean follow-up time across the cohort was 1465.84 days (SD 1006.45), with Northern Ontario patients followed for longer periods (1671.75 days) than those in Southern Ontario (1424.93 days). The percent of days covered (PDC) was similar across regions, with a mean of 48.47% (SD 36.49), slightly higher in Southern Ontario (48.86%) than in Northern Ontario (46.50%). Buprenorphine/naloxone PDC was higher in Northern Ontario (448.90 days) than in Southern Ontario (262.35 days), with an SMD of 0.301. Methadone PDC was greater in Southern Ontario (243.17 days) than in Northern Ontario (153.95 days). The results are presented in Table 2.

**Table 2:** OAT, comorbidities and health service use among cohort patients

	Ontario total	Southern Ontario	Northern Ontario	SMD
	N = 76,890	n = 64,623 (84.0%)	n = 12,267 (16.0%)	
OAT starting medications, *n (%)*				0.400
Buprenorphine/naloxone	36,002 (42.1)	28,647 (45.3)	7355 (61.9)	
Methadone	38,943 (44.8)	34,370 (54.7)	4573 (38.1)	
Other	1945	1606	339	
Frist OAT Attempt days retained, *mean (SD)*	166.04 (297.25)	172.45 (301.37)	133.77 (273.32)	0.134
Buprenorphine/naloxone	112.87 (248.91)	94.25 (208.07)	206.57 (381.24)	0.366
Methadone	231.50 (416.64)	243.05 (428.96)	173.35 (342.28)	0.18
Follow up time in days*, mean (SD)*	1465.84 (1006.45)	1424.93 (1008.08)	1671.75 (972.49)	0.249
Percent of days covered, *mean (SD)*	48.47 (36.49)	48.86 (36.48)	46.50 (36.51)	0.070
Buprenorphine/naloxone	293.28 (574.14)	262.35 (543.01)	448.90 (689.66)	0.301
Methadone	228.38 (396.97)	243.17 (410.86)	153.95 (307.35)	0.246
Mental Disorder *n (%)*	34,051 (68.0)	29,458 (70.8)	4593 (54.4)	0.343
Schizophrenia *n (%)*	4305 (5.6)	3688 (5.7)	617 (5.0)	0.035
Bipolar disorder *n (%)*	3987 (8.0)	3525 (8.5)	462 (5.5)	0.336
Alcohol Use Disorder, *n (%)*	6879 (9.0)	5353 (8.3)	1526 (12.7)	0.144
Chronic Pain n (%)	56,657 (73.3)	48,430 (75.1)	8227 (64.2)	0.239
HIV n (%)	606 (0.8)	505 (0.8)	101 (0.8)	0.016
All -cause ED visits	12.82 (35.11)	12.65 (35.41)	13.71 (33.45)	0.031
*mean per patient per year (SD)*				
Mental Health and Substance-related ED visits	8.53 (16.36)	8.25 (16.38)	10.00 (16.17)	0.107
*mean per patient per year (SD)*				
All cause Hospital admissions	1.38 (2.93)	1.35 (2.90)	1.53 (3.10)	0.060
*mean per patient per year (SD)*				
Mental Health and Substance-related Hospital Admissions	1.27 (2.91)	1.24 (2.88)	1.43 (3.06)	0.065
*mean per patient per year (SD)*				

*n = number

*mean = mean per year

*SD = standard deviation

*SMD = standardized mean difference

The prevalence of mental health disorders varied significantly between the regions. Overall, 70.8% of people living in Southern Ontario had a diagnosed mental disorder and 54.4% in northern Ontario. 5.6% were diagnosed with schizophrenia, with a slightly higher proportion in Southern Ontario (5.7%) compared to Northern Ontario (5.0%). Bipolar disorder was reported in 3987 people (8.0%), notably more prevalent in Southern Ontario (8.5%) than in Northern Ontario (5.5%). Alcohol use disorder was present in 6879 (9.0%) with differences across both regions (8.3% in Southern Ontario and 12.7% in Northern Ontario). Chronic pain was more common in Southern Ontario 75.1% than Northern Ontario 64.2%. 0.8% of the cohort is diagnosed with HIV and there were no regional differences. The mean all-cause emergency department (ED) visits per patient per year was 12.65 (standard deviation (SD) 35.41) in Southern Ontario versus 13.71 (33.45) in Northern Ontario. The mean ED visits per patient per year for reasons mental health and substance use was 8.25 (SD 16.38) in Southern Ontario and 10.00 (SD 16.71) in Northern Ontario. The mean of all-cause hospital admissions per patient per year was 1.35 (SD 2.90) in Southern Ontario and 1.53 (SD 3.10) in Northern Ontario. The mean hospital admissions per patient per year for reasons mental health and substance use was 1.24 (SD 2.88) in Southern Ontario and 1.43 (SD 3.06) in Northern Ontario. Results are presented in Table 2.

### OAT engagement in Ontario

The majority (56.1%) of people in OAT began treatment after 2014, however, a substantial proportion (43.9%) were long-term users who started before 2014 and remained in treatment throughout the follow-up period. We also found that in Southern Ontario, methadone was the more common initial treatment, with 53.2% starting on it. Conversely, Northern Ontario buprenorphine/naloxone was the more common starting medication (62.7%). These differences may reflect varying prescribing practices, healthcare access, or patient preferences across regions.

### Demographic differences in Ontario

**Fig. 2:**
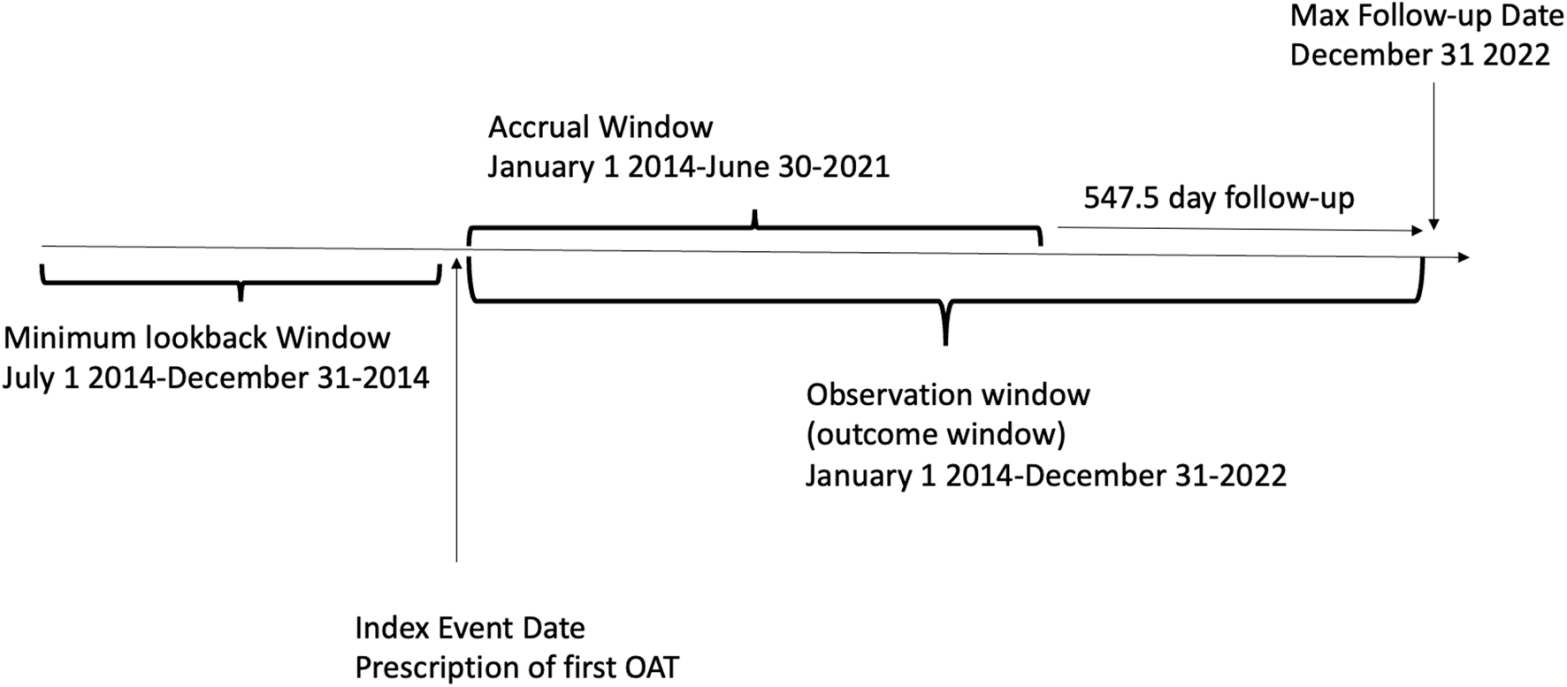
Cohort follow-up

The data highlights key demographic, geographic, and socioeconomic differences between OAT patients in Northern and Southern Ontario. The patients in the North were significantly younger, with a slightly higher proportion of females in Northern Ontario (44.3%), had a lower average quintile and live in rural and remote all factors creating additional barriers to healthcare access. Homelessness affected 26.6% of the cohort, which reflects a vulnerable population in need of integrated social and healthcare services. Our cohort demonstrates significant geographic, demographic, and socioeconomic disparities between Northern and Southern Ontario, highlighting the need for region-specific approaches to OAT delivery, particularly in rural and remote areas (Fig. 2).

### OAT retention

**Fig. 3:**
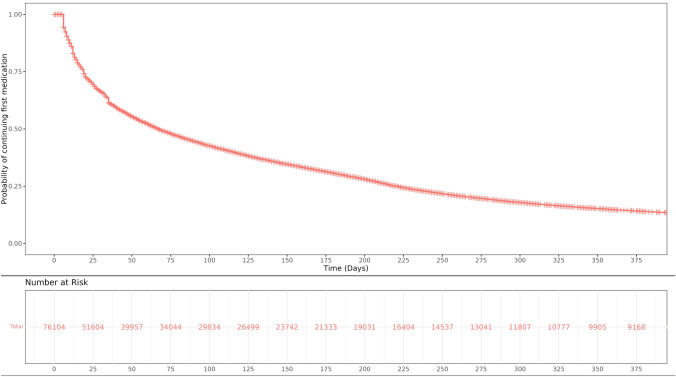
Probability of patient retention in days

We found that the probability of OAT retention at 30 the day mark in this cohort is approximately 50%, at 90 days it’s about 45 and at one year, it’s approximately 25%. Results are displayed in Fig. 3.

## Strengths and limitations

The main strengths are event-level data for patient medication use, health care services resource use and physician and geographic variation in practice patterns at a population-level across different regions of Ontario. The use of multiple health administrative databases linked through unique identifiers provides a comprehensive view of individuals' healthcare utilization. Such linkage enables a detailed analysis of health service use and treatment patterns. The cohort allows for observational studies that mimic randomized controlled trials (RCTs) at a population level. By using existing data and applying careful eligibility criteria and treatment definitions, researchers can assess the effectiveness of interventions and healthcare system changes in a real-world setting. The cohort covers both Southern and Northern Ontario, offering insights into regional disparities in healthcare access and treatment. The north-south comparison in Ontario is particularly valuable for other jurisdictions because southern Ontario has a high concentration of densely populated urban areas, while northern Ontario is characterized by less populated and more rural regions. These distinctions are crucial for countries with a mix of highly dense urban centers and lower-density rural areas, as they highlight how OAT access, delivery, and outcomes may vary depending on population density and geographic context. The inclusion of socioeconomic data, allows for a nuanced understanding of how socio-economic factors impact treatment outcomes and healthcare utilization.

While the cohort provides detailed data for Ontario, the findings may not be directly generalizable to other regions with different healthcare systems or population characteristics. Additionally, although the observational approach can emulate RCTs, it still has limitations such as potential biases in treatment allocation and confounding factors that are not always fully controlled. The reliance on existing data may also limit the ability to address all potential variables influencing outcomes. The accuracy of the results also depends on the completeness and correctness of the data within the administrative databases. While the cohort covers diverse regions, the concentration of OAT services and interventions in Southern and urban areas may result in less detailed information about rural and Northern regions. This uneven distribution could affect the interpretation of findings. The cohort’s reliance on administrative data may limit the depth of understanding of patient experiences.

## Collaboration and data sharing

The dataset from this study is held securely in coded form at ICES. While legal data sharing agreements between ICES and data providers (e.g., healthcare organizations and government) prohibit ICES from making the dataset publicly available, access may be granted to those who meet pre-specified criteria for confidential access, available at www.ices.on.ca/DAS (email: das@ices.on.ca). The full dataset creation plan and underlying analytic code are available from the authors upon request, understanding that the computer programs may rely upon coding templates or macros that are unique to ICES and are therefore either inaccessible or may require modification. Any inquiries or clarifications regarding the project can be directed via email to Dr. David Marsh at dmarsh@nosm.ca.

## Conclusion

The OAT cohort in Ontario, Canada, provides valuable insights into OAT at a population level. It is a unique resource for evaluating health system patterns and outcomes for PWOUD over time. With a set of clinical, social, and demographic variables, the cohort allows for a comprehensive evaluation of various exposures and outcomes, making it a high-priority area of interest in the field of health and social policy and services at all levels of government.
